# Patient Characteristics in the Recording Courses of Vascular Diseases (Reccord) Registry: Comparison with the Voyager Pad Endovascular Cohort

**DOI:** 10.3390/jcdd10030115

**Published:** 2023-03-10

**Authors:** Michael Czihal, Nasser Malyar, Jürgen Stausberg, Ulrich Hoffmann

**Affiliations:** 1 Division of Vascular Medicine, Medical Clinic and Policlinic IV, Hospital of the Ludwig-Maximilians-University, Ziemssenstraße 5, 80336 Munich, Germany; 2 Department of Cardiology I—Coronary and Peripheral Vascular Disease, Heart Failure, University Hospital, 48149 Muenster, Germany; 3 Independent Researcher, 45276 Essen, Germany

**Keywords:** peripheral arterial disease, critical limb threatening ischemia, endovascular revascularization, registry, randomized controlled trial

## Abstract

Background: To compare the characteristics of a “real world” population included in a prospective registry to patients enrolled in a randomized, controlled trial (RCT) after endovascular revascularization (EVR) for symptomatic peripheral artery disease (PAD). Methods: The RECcording COurses of vasculaR Diseases (RECCORD) registry is an observational registry prospectively recruiting patients undergoing EVR for symptomatic PAD in Germany. VOYAGER PAD was an RCT which demonstrated the superiority of rivaroxaban and aspirin versus aspirin to reduce major cardiac and ischemic limb events following infrainguinal revascularization for symptomatic PAD. For this exploratory analysis, the clinical characteristics of 2.498 patients enrolled in RECCORD and of 4.293 patients from VOYAGER PAD who underwent EVR were compared. Results: The rate of patients aged ≥ 75 years was considerably higher in the registry (37.7 vs. 22.5%). More patients in the registry had undergone previous EVR (50.7 vs. 38.7%) or suffered from critical limb threatening ischemia (24.3 vs. 19.5%). Registry patients were more commonly active smokers (51.8 vs. 33.6%), but less frequently suffered from diabetes mellitus (36.4 vs. 44.7%). While statins (70.5 vs. 81.7%) were less frequently used, antiproliferative catheter technologies (45.6 vs. 31.4%) and postinterventional dual antiplatelet therapy (64.5 vs. 53.6%) were more commonly applied in the registry. Conclusions: There were many similarities but some clinically meaningful differences in clinical characteristics between PAD patients who underwent EVR and were included in a nationwide registry and PAD patients from the VOYAGER PAD trial.

## 1. Introduction

Lower extremity peripheral arterial disease (PAD) affects approximately 50 million inhabitants in Europe [1]. PAD patients are at high risk for developing myocardial infarction, stroke, lower extremity ischemic events and cardiovascular death. Endovascular revascularization (EVR) procedures of the lower extremity arteries are increasingly used for the treatment of PAD, aiming at improving the quality of life in symptomatic patients and saving limbs in patients suffering from critical limb threatening ischemia (CLTI) [2]. Despite substantial advances in medical treatment over the past decades, the cardiovascular risk of PAD patients after having undergone EVR remains high, and the risk of acute ischemic limb events is substantially increased [3]. 

Unfortunately, until recently there has been an eminent lack of evidence on the efficacy and safety of antithrombotic treatment after EVR in PAD, making treatment decisions subject to individual physicians’ judgements based on patients’ and procedure characteristics [4]. The recently published VOYAGER PAD trial led to a paradigm change in antithrombotic treatment after revascularization of PAD, as it demonstrated a net clinical benefit of a dual antithrombotic regimen (ASA and low dose Rivaroxaban) over ASA and placebo [5]. It is of great clinical importance to identify factors potentially interfering with the translation of these pivotal trial results into broad daily clinical practice. Therefore, this study aimed to compare the clinical characteristics of patients prospectively recruited into a large prospective German EVR registry (RECording COurses of vasculaR Diseases, RECCORD [6]) with those of the patients included in VOYAGER PAD.

## 2. Materials and Methods

The RECcording COurses of vasculaR Diseases (RECCORD) registry was established by the German Society of Angiology—Society for Vascular Medicine in order to address the lack of contemporary real-world data regarding the current practice of medical and interventional care in vascular patients (list of RECCORD Registry Collaborators in the Appendix A). The RECCORD study protocol was approved by the Ethics Committee of the Ludwig-Maximilians-University, Munich, Germany. The study protocol, the clinical characteristics of the first 1.000 patients and the current practice of EVR in different anatomic regions of the lower limbs have been published previously [6,7,8]. Having provided written informed consent, all patients with symptomatic lower extremity PAD (lesions located from the aorto-iliac bifurcation to the distal crural arteries) undergoing EVR can be included, whereas there are no dedicated exclusion criteria. RECCORD collects data regarding the diagnosis of PAD (based on ICD-10-codes) and of the endovascular procedures (based on OPS-codes) together with 84 items regarding anthropometry, medical history including previous revascularization, cardiovascular comorbidities and risk profile, medication, PAD symptoms including walking distance, hemodynamic situation (ankle brachial index, ABI), and quality of life. 

VOYAGER PAD was a randomized, controlled, double-blind trial which compared the combination therapy of ASA + low dose Rivaroxaban compared to ASA + placebo after infrainguinal revascularization for PAD [5]. The study design, including in- and exclusion criteria and the principle outcomes of the VOYAGER PAD, have been published elsewhere [9]. Briefly, patients aged ≥ 50 years with moderate to severe, symptomatic lower extremity PAD were eligible for randomization after successful arterial revascularization (either endovascular or surgical) below the inguinal ligament. PAD-related exclusion criteria included asymptomatic or only mildly symptomatic PAD, prior revascularization of the index leg within 10 day of the qualifying revascularization, acute limb ischemia, and major tissue loss. VOYAGER PAD revealed a significantly lower incidence of the composite primary outcome of acute limb ischemia, major amputation for vascular causes, myocardial infarction, ischemic stroke, or death from cardiovascular causes in the combined treatment group compared to patients treated with aspirin alone.

For the current analysis, 2.498 subjects enrolled in RECCORD between February 2019 and September 2020 and 4.293 patients from the VOYAGER PAD cohort who had undergone EVR (including hybrid procedures) and were recruited between August 2015 and January 2018 were compared. Aggregate data of the VOYAGER PAD endovascular cohort, including demographic information, clinical and procedural characteristics as well as cardiovascular medication, were provided by the VOYAGER PAD steering committee. Based on these aggregate data of the RCT, matching variables of the RECCORD database were exported. If necessary, the categorization of nominal and ordinal variables of the RECCORD cohort was modified for proper comparison with the aggregated RCT data. Analysed parameters included PAD severity (claudication vs. critical limb ischemia), the type of endovascular procedure, the history of prior lower limb revascularization (either endovascular or surgical) or amputation, a cardiovascular risk profile including severe chronic renal insufficiency (defined as eGFR < 30 mL/min/1.73 m^2^), cardiovascular comorbidities including coronary artery disease (CAD) and cerebrovascular disease (CVD), ABI of the index leg prior to EVR, and cardiovascular medication (single or dual antiplatelet treatment, statin treatment, ACE inhibitors/AT1-antagonists and ß-Blockers). The definition of CLTI in the RECCORD-cohort was based on ICD-10-coding (I70.23, I70.24, I70.25), whereas in VOYAGER PAD a diagnosis of CLTI was assigned to patients with PAD fulfilling established clinical (rest pain, ulcers, gangrene) and hemodynamic criteria (ankle pressure ≤ 50 mmHg and rest pain; ankle pressure ≤ 70 mmHg and tissue loss) [10]. CVD was characterized as a composite of prior stroke, history of carotid revascularization or carotid artery stenosis > 50% in VOYAGER PAD, but was not further specified in RECCORD. 

For all comparisons regarding cardiovascular medication we used VOYAGER PAD baseline data (representing any medication started 30 days before and after randomization). For the comparison of the rates of antihypertensive and statin treatment, we used RECCORD baseline data (obtained prior EVR). For the comparison of the rates of dual antiplatelet treatment we relied on the documented drug information covering the time period between the RECCORD baseline visit (prior EVR) and a maximum of 14 days following the index intervention. Due to limited items in the RECCORD database, we were not able to provide exact data on the type of single antiplatelet therapy (ASA vs. clopidogrel). 

As the analysis relied on aggregate data, we performed exploratory analyses of differences between both cohorts, without testing for statistical significance. Continuous data are presented as mean ± standard deviation. Categorical data are given as absolute numbers (percentages). 

## 3. Results

The comparison between both cohorts is outlined in Table 1. Females represented 34.1% of patients in the registry and 28.8% of patients in the trial (male to female ratio 1.93 in the registry; 2.47 in the RCT). The mean patient’s age was somewhat higher in registry patients (70.3 ± 10.4 years) compared to the RCT patients (67.6 ± 8.6 years). Correspondingly, the rate of patients aged ≥ 75 years was considerably higher in the registry (37.7% vs. 22.5%). The proportions of White, Black/ African American and Asian ethnicities were 76.3%, 3.2%, and 18.5% in the RCT. RECCORD did not collect data on the distribution of ethnic groups.

More than half of the registry patients (50.7%) had previously undergone any limb revascularization (previous EVR in 47.2%, previous bypass surgery in 12.9%). In the RCT, the respective rates for any previous revascularization, previous EVR and previous bypass surgery were somewhat lower (38.7%, 35.2%, 6.6%). By contrast, the rate of any prior amputation was lower in the registry (4.0 vs. 6.0%). Almost every fourth of the registry patients (24.3%) suffered from CLTI, compared to nearly every fifth patient in the RCT (19.5%). However, the mean ABI of the index leg was higher in the registry (0.65 ± 0.3) than in the RCT (0.57 ± 0.18). Due to differences in categorization, lesion lengths (registry: <10 cm, 10–20 cm, RCT: < 5 cm, 5–14.9 cm, >15 cm) were not directly comparable between the cohorts. Antiproliferative technologies (drug coating balloons, drug eluting stents) were applied in 1.138 registry patients (45.6%) and in 1.330 RCT patients (31.4%). More precise information regarding the lesion location and type of procedure was available for the registry cohort but not for the RCT dataset, and therefore comparisons were not feasible.

Although the mean weight and mean BMI were very similar between both groups, there was a higher rate of diabetes mellitus in the RCT (44.7%) compared to the registry (36.4%). Conversely, many more patients in the registry than in the RCT were active smokers (51.8 vs. 33.6%). We further observed a higher percentage of patients with severely impaired kidney function in the registry (3.0%) than in the RCT (0.8%). Otherwise, there were no remarkable differences regarding the presence of classic cardiovascular risk factors. While the reported rate of CAD and a history of heart failure were similar in both cohorts, the reported frequency of CVD was lower in the registry (15.9%) compared to the RCT (32.8%).

Dual antiplatelet treatment was commenced in 2.259 RCT patients (53.6%), compared to roughly two thirds of registry patients (*n* = 1.611, 64.5%). Comparisons regarding cardiovascular medication are given in Table 2. Statins as well as ACE-inhibitors/ARB-antagonists were less commonly taken by registry patients (70.5% and 58.1%) than by RCT patients (81.7% and 65.4%). 

## 4. Discussion

This exploratory analysis elucidated many similarities but some important differences in clinical characteristics between PAD patients who underwent EVR within routine care and were included in a nationwide registry, and patients randomized to EVR in the VOYAGER PAD trial. The most important differences included a considerably higher rate of patients aged ≥ 75 years (registry 37.7% vs. RCT 22.5%), and of patients with a history of previous revascularization procedures (approaching 50% in patients treated during routine clinical practice) in the registry cohort. Moreover, the proportion of CLTI patients and of patients with advanced chronic kidney disease was somewhat higher in the registry compared to the RCT.

The rate of major adverse limb events (MALE, defined as composite of acute limb ischemia, amputation and unplanned index limb revascularization) in the VOYAGER PAD endovascular cohort was estimated to be as high as 30% after 3.5 years, with 23.5% represented by unplanned index limb revascularization [11]. Recently published health claims-based data underscored a substantial risk of MALE after EVR, with a rate of hospitalization for MALE of 12.9% in 400.000 patients who underwent EVR of PAD in the US (median follow-up: 2.7 years) [3]. CLTI and advanced age, both somewhat more common in the registry compared to the RCT, have been shown to be negatively associated with extremity outcomes (e.g., amputation free survival) in PAD cohorts [12,13,14,15]. The same is true for advanced chronic kidney disease, which was three times more common in the registry, although absolute numbers were low [14,16]. Repeat EVR after previous revascularization procedures may also be associated with worse outcomes [17,18]. In the RECCORD registry, almost every second patient had previously undergone an endovascular procedure. 

Thus, the cohort comparison of RECCORD and the RCT indicates an ischemic risk profile which could be worse in patients treated within routine clinical practice, underlining the need for effective treatment strategies in order to avoid repeated ischemic events and/or target lesion revascularization. Results of a comparison of the Danish Vascular Registry and the overall cohort of the VOYAGER PAD trial (including surgically revascularized patients) point in the same direction, with higher mean age and substantially higher rates of critical limb ischemia and current smoking in the registry but a lower percentage of patients under statin treatment [19]. It is of interest in this context that the rate of patients under guideline-recommended pharmacotherapy was lower in the RECCORD registry (e.g., statin therapy 70%) compared to the RCT (e.g., statin therapy 80%), but higher than in contemporary analyses of routine ambulatory care (e.g., statin therapy in 50% of patients in a health claims based study by Rammos et al.) [20].

Drug eluting technologies (balloon catheters, stents) were applied more frequently in registry patients, compared to the RCT. This difference probably reflects the international variation of endovascular practice, which may be influenced by reimbursement issues. Furthermore, study periods were different (the registry started shortly after recruitment for the RCT had been completed) and endovascular practice in both study populations with regard to the use of drug eluting technologies may have been influenced by a much-acclaimed publication from 2018, suggesting an increased mortality risk with paclitaxel-based devices [21], and several subsequent studies which contradicted this study’s findings [11,22,23]. 

It is common clinical practice to prolong dual antiplatelet therapy after the application of drug coated devices [24], and in the registry two out of three patients were put on dual antiplatelet therapy after EVR. However, due to a lack of scientific evidence on the clinical benefit of dual antiplatelet therapy after EVR, and considering the results of VOYAGER PAD, dual antithrombotic therapy rather than dual antiplatelet treatment is favored after infrainguinal EVR by current European expert recommendations [25]. 

In VOYAGER PAD there was an increased risk for bleeding events under dual antithrombotic treatment (low dose rivaroxaban and aspirin) compared to aspirin monotherapy, but no excess of intracranial and fatal bleeding [5]. Short term clopidogrel did not further increase the bleeding risk [26], but the impact of patient’s characteristics on bleeding complications has not been analysed in detail so far. It is well known, however, that the risk factors of ischemic events which we found more commonly in the RECCORD registry (advanced age and CLTI) are also important risk factors for bleeding events under single or dual antiplatelet therapy [27,28]. Both factors were recently confirmed as independent predictors of bleeding complications in a health claims data-based analysis of patients who were hospitalized for symptomatic PAD [29]. Given these data, the cohort comparison of RECCORD and the RCT also indicates a bleeding risk profile which could be rather worse in patients treated within routine clinical practice.

Patient registries are promoted to close the efficacy–effectiveness gap between evidence from RCTs and outcomes that could be achieved in daily health care [30]. The need to control most of the confounders typically leads to a homogeneous cohort of patients with lower risks in RCTs. The comparison between both cohorts highlights this situation with lower frequencies of some—but not all—relevant risk factors in the VOYAGER PAD trial. Furthermore, protocol-based procedures of an RCT result in an optimized therapy, as indicated here by the more frequent guideline-recommended pharmacotherapy in the VOYAGER PAD trial. In view of the higher rate of female patients in RECCORD, the comparison also underpins the observation that women are underrepresented in the enrolment of RCTs [31]. Registry-based RCTs might be an option to combine the advantages of both designs and to better control for the efficacy–effectiveness gap [32].

The main limitation of this study is that it is an exploratory analysis based on aggregate and not on single patient data. Patients in the RCT may have been characterized more deeply, which, for example, could explain the higher rate of diabetes mellitus in the RCT. Due to differences in categorizations, we were not able to compare some variables such as ethnic origin, exact data on the drugs used for single antiplatelet therapy, and lesion lengths of the target lesions. Given the lack of information on the ethnic descent of patients in RECCORD, a comparison with the subgroup of German patients who received endovascular treatment within the RCT might be of interest. However, data on the German subgroups of the VOAYAGER PAD are not yet available. Different definitions of CLTI and CVD may have affected the observed frequency of these conditions in the two cohorts, and the different timings of medication recording could have impacted the observed rates of medical treatments, such as dual antiplatelet and statin therapy. Finally, in contrast to the RCT the registry also included patients with iliac artery involvement.

Nevertheless, our study has implications for clinical practice. We observed no serious differences in demographic characteristics, cardiovascular disease/risk profiles and treatment that could prohibit the extrapolation of the RCT results on the registry. Noteworthy was a higher rate of elderly patients and of patients with CLTI and/or a history of previous revascularization in the registry indicating worse risk profiles for both ischemic as well as bleeding events in routine clinical practice. This should give reason for very careful patient selection for intensified antithrombotic treatment as well as a structured and intensive follow up after EVR. Further analyses of the RECCORD registry will elucidate periprocedural bleeding complications and intermediate follow-up data of patients treated within routine clinical practice.

## Figures and Tables

**Table 1:** Comparison of demographic and clinical characteristics between patients treated in the registry and the RCT.

	RECCORD *n* = 2.498	VOYAGER PAD Endovascular Cohort *n* = 4.293
Age, years (mean ± SD)	70.3 ± 10.4	67.6 ± 8.6
Age ≥ 75 years, *n* (%)	942 (37.7)	966 (22.5)
Female sex, *n* (%)	852 (34.1)	1.238 (28.8)
Weight, kg (mean ± SD)	78.5 ± 17.1	76.2 ± 16.5
BMI, kg/m^2^ (mean ± SD)	26.0 ± 4.7	26.6 ± 4.7
Diabetes mellitus, *n* (%)	909 (36.4)	1.920 (44.7)
Arterial hypertension, *n* (%)	2.036 (81.5)	3.517 (81.9)
Dyslipidemia, *n* (%)	1.715 (68.7)	2.766 (64.4)
Current smoking, *n* (%)	1.294 (51.8)	1.442 (33.6)
eGFR < 30 mL/min, *n* (%)	75 (3.0)	36 (0.8)
Known CVD, *n* (%)	396 (15.9)	1.409 (32.8)
Known CAD, *n* (%)	834 (33.4)	1.387 (32.3)
History of heart failure, *n* (%)	208 (8.3)	320 (7.5)
Index ABI (mean ± SD)	0.65 ± 0.3	0.57 ± 0.18
Critical limb ischemia, *n* (%)	607 (24.3)	837 (19.5)
Prior amputation, *n* (%)	101 (4.0)	260 (6.0)
Prior limb revascularization, *n* (%)	1.267 (50.7)	1.663 (38.7)
Prior surgical bypass, *n* (%)	323 (12.9)	283 (6.6)
Prior endovascular revascularization, *n* (%)	1.183 (47.4)	1.510 (35.2)

**Table 2:** Comparison of drug therapies between patients treated by EVR in the registry and the RCT.

	RECCORD *n* = 2.498	VOYAGER PAD Endovascular Cohort *n* = 4.293
Dual antiplatelet therapy, *n* (%)	1.611 (64.5)	2.299 (53.6)
Statins, *n* (%)	1.760 (70.5)	3.509 (81.7)
ACE-inhibitors/ARB-antagonists, *n* (%)	1.451 (58.1)	2.806 (65.4)
ß-Blockers, *n* (%)	1.226 (49.1)	1.876 (43.7)

## Data Availability

The data that support the findings of this study are available from the corresponding author upon reasonable request.

## Appendix A. RECCORD Registry Collaborators

Dr. Jawed Arjumand, AGAPLESION BETHESDA Krankenhaus gGmbH, Wuppertal, Germany; Jens Böhme, Ambulantes Zentrum für Herz- und Gefäßkrankheiten Ostbrandenburg, Frankfurt/Oder, Germany; Prof. Dr. med. Christian Erbel, Universitätsklinikum Heidelberg, Heidelberg, Germany; Matthias Fischer, Gefäßzentrum Berlin Helle-Mitte, Berlin, Germany; Prof. Dr. Wulf Ito, Klinikverbund Kempten-Oberallgäu GmbH, Immenstadt, Germany; PD Dr. Christoph Kalka, Marienhospital Brühl, Brühl, Germany; Prof. Dr. Grigorios Korosoglou, GRN-Klinik Weinheim, Weinheim, Germany; Dr. Ralf Langhoff, Sankt Gertrauden-Krankenhaus GmbH, Akad. Lehrkrankenhaus der Charité—Universitäts-medizin, Berlin, Germany; Dr. Michael Lichtenberg, Klinikum Hochsauerland, Arnsberg, Germany; Prof. Dr. Sebastian Schellong, Städtisches Krankenhaus Dresden, Dresden, Germany; Jörg Schwuchow, OGD Ostprignitz-Ruppiner Gesundheitsdienste GmbH, Neuruppin, Germany; Jens Stegemann, Evangelisches Krankenhaus Königin Elisabeth Herzberge gGmbH, Berlin, Germany; Prof. Dr. Tareq Ibrahim, TUM Klinikum rechts der Isar, München, Germany.
